# The Joint Association of Daily Rest Periods and Sleep Duration with Worker Health and Productivity: A Cross-Sectional Web Survey of Japanese Daytime Workers

**DOI:** 10.3390/ijerph191711143

**Published:** 2022-09-05

**Authors:** Hiroki Ikeda, Tomohide Kubo, Shuhei Izawa, Nanako Nakamura-Taira, Toru Yoshikawa, Rie Akamatsu

**Affiliations:** 1 National Institute of Occupational Safety and Health, Japan Organization of Occupational Health and Safety, Kawasaki 214-8585, Japan; 2 Department of Psychology, Faculty of Letters, Chuo University, Tokyo 192-0393, Japan; 3 Natural Science Division, Faculty of Core Research, Ochanomizu University, Tokyo 112-8610, Japan

**Keywords:** daily rest period, sleep duration, quick return, mental health, sleep quality, presenteeism

## Abstract

A daily rest period (DRP) is a daily inter-work interval that contains sleep opportunity. This study investigates the joint association of DRP and sleep duration with worker health and productivity. A total of 13,306 Japanese daytime workers participated in this web-based cross-sectional survey. Participants reported on their DRPs and sleep duration; moreover, sleep difficulties, mental health, and presenteeism were assessed by the standardized questionnaires. The participants were divided into 10 groups based on their DRPs and sleep duration. Logistic regression analyses showed that the combination of quick return (QR: DRP of <11 h) and short sleep duration (<6 h) was found to be significantly associated with sleep difficulties (odds ratio [OR] = 4.45, 95% confidence interval [CI] = 2.83–7.01), poor mental health (OR = 3.04, 95% CI = 1.79–5.15), and presenteeism (OR = 2.35, 95% CI = 1.47–3.77) compared with the reference group (the combination of adequate DRP [15 h] and a normal sleep duration [≥6 h]). The combination of QR and normal sleep duration or adequate DRP and short sleep duration was significantly associated with high ORs for the outcomes. QR, short sleep duration, or both negatively affect worker health and productivity.

## 1. Introduction

Daily rest period (DRP) refers to the daily inter-work interval between the end of one workday and the beginning of the subsequent workday. The European Union (EU) working time directive states that EU workers have the right to take “a minimum daily rest period of 11 consecutive hours every 24 h” [1]. The Japanese “work interval system” requires employers to ensure workers are given a certain interval of hours from the end of one workday to the start of the next (i.e., DRP) [2]. DRP can include a sleep opportunity (the amount of time the person can use for sleep), leisure time, and other non-work time activities. Therefore, as workers with short DRPs tend to have short sleep durations [3], a DRP extension may increase their sleep duration [4]. Short DRPs, especially those of less than 11 h, referred to as a quick return to work (QR), have been found to associate with negative outcomes in worker health and productivity such as poor sleep quality [3,4,5], stress [6,7], fatigue [8,9,10], and absenteeism [11,12]. It is demonstrated that ensuring an adequate DRP may be important to achieve good worker health and productivity.

Sleep duration, which is part of the DRP, can also affect aspects of worker health and productivity, such as sleep quality [13], mental health [14], presenteeism (inadequate work performance due to illness) [15,16], and absenteeism [16]. Therefore, both DRP and sleep duration may be associated with worker health and productivity. However, the joint association of DRP and sleep duration with worker health and productivity is unclear. Although DRP has been found to correlate with both sleep duration and leisure time, its correlation with sleep duration was significantly weaker than that with leisure time [3]. Therefore, some workers may have a certain sleep duration despite having short DRPs as they sacrifice leisure time to prioritize sleep durations. Of workers with a short DRP, those who have a certain sleep duration may have fewer negative effects on their health and productivity than those who have short sleep duration. Conversely, some workers may have short sleep durations despite having sufficient DRPs as they sacrifice sleep to prioritize leisure time. Of workers who have a DRP considered adequate based on the EU working time directive or the Japanese “work interval system”, those who have short sleep durations may experience worse effects on their health and productivity than those who have normal sleep durations.

This study investigated the joint association of DRP and sleep duration with worker health and productivity. Sleep quality and mental health were measured as the health-related outcomes and presenteeism as the work productivity-related outcome. These outcomes were selected based on evidence that they are associated with both a short DRP (or long working hours, which is inextricably linked to a short DRP) [3,4,5,17,18] and short sleep duration [13,14,15]. In Japan, survey data indicated that 53.3% of employees had experienced something that caused their anxiety, worry, or distress in their current work or occupational life within the past year, and 10.1% of establishments reported that employees had been absent from work or resigned because of mental health problems [19]. The Japanese government, therefore, encourages the promotion of mental health in the workplace [20]. To do this successfully, it is important to understand the association between DRP, sleep duration, and workers’ mental health.

This study was driven by three hypotheses:

**Hypothesis 1** **(H1).** *The combination of a short DRP and short sleep duration is associated with increased odds of sleep difficulty, poor mental health, and presenteeism compared with the combination of an adequate DRP and normal sleep duration, and these odds are the highest among any other combination of DRP and sleep duration.*

**Hypothesis 2** **(H2).** *The combination of a short DRP and normal sleep duration is associated with increased odds of sleep difficulty, poor mental health, and presenteeism compared with the combination of an adequate DRP and normal sleep duration.*

**Hypothesis 3** **(H3).** *The combination of an adequate DRP and short sleep duration is associated with increased odds of sleep difficulty, poor mental health, and presenteeism compared with the combination of an adequate DRP and normal sleep duration.*

As shiftwork and night work can disturb circadian rhythms and influence worker health and productivity, this study only focused on daytime non-shift workers.

## 2. Materials and Methods

### 2.1. Survey and Sampling

“Web-based Longitudinal study of the Work Environment and daily Lifestyle (WELWEL)” is an ongoing large-scale study of workers in Japan that investigates the associations between daily life self-care behaviors, such as stress management, social support, sleeping habits, eating patterns, and exercise; work environment improvements, such as the readjustment of work tasks and work skill training opportunities; and mental health outcomes in representative Japanese employee samples. The first survey was conducted in February 2022. Data collection was performed online and was outsourced to a research company. Japanese workers who had registered were sampled based on the distribution ratios for all Japanese employees [21]: gender, age (20–29, 30–39, 40–49, and 50–59 years), and 20 industry types, such as construction and manufacturing. The following were excluded from the sample: (a) agriculture, forestry, fishery, mining, and quarry workers; (b) self-employed workers and company executives; (c) those working a second job; (d) those working less than 20 h per week; and (e) those who responded inappropriately to the instructional manipulation check [22] and/or had an extremely short response time (≤10 min). Exclusion criteria (a) and (b) were based on our previous study [23]. The research company sent invitations to participate to 421,825 registrants, and 115,094 accessed the survey website produced by the company. The first 20,000 registrants who met the distribution ratios for Japanese employees and exclusion criteria, participated in the survey.

Informed consent was obtained from all participants, and this study was approved by the Research Ethics Committee of the National Institute of Occupational Safety and Health, Japan (2021N-1-19).

### 2.2. Measurements

The sleep duration question gathered information on how many hours the participants normally slept, and the start and end of working hours question gathered data on the average work start and end times in the previous month, with the answers being given in 5-min intervals. The DRP was then calculated as the interval from the end of the working hours to the beginning of the working hours.

Sleep difficulties and mental health were assessed using the Athens Insomnia Scale (AIS) and the K6 scale, respectively. The AIS, a self-administered questionnaire, which is based on the International Classification of Diseases 10th criteria, has been widely employed to evaluate sleep difficulty intensities [24]. This study used the Japanese version of AIS, which has been proven to exhibit sufficient reliability and validity [25]. The AIS comprises eight items asking about sleep disturbances and daytime dysfunctions in the preceding month. Each item is rated on a 4-point Likert scale. The AIS score is the sum of the item scores (range: 0–24), with higher scores indicating greater sleep difficulties. Scores of six points or more are considered to indicate pathological insomnia [25]; therefore, a cutoff value of six points was used to identify those experiencing sleep difficulties. Cronbach’s alpha coefficient for the scale was 0.84 in the current study. This study also used the Japanese version of the K6 scale [26] to assess mental health, which has been proven to exhibit acceptable reliability and validity [27]. The K6 scale, a self-administered questionnaire, has six items that ask about the psychological distress symptoms experienced in the previous 30 days, with each item being rated on a 5-point Likert scale. The overall K6 score is the sum of the item scores (range: 0–24), with higher scores indicating poorer mental health. A score of 13 points or more indicates severe mental illness and hence poor mental health [28]; therefore, 13 was used as the cutoff value for mental health quality. The Cronbach’s alpha coefficient for the scale was 0.93 in the current study.

One item from the Japanese version [29] of the World Health Organization Health and Work Performance Questionnaire (WHO-HPQ) short form [30,31], a self-administered questionnaire, was used to assess presenteeism. The question asked the participants to rank their job performances over the previous 4 weeks (28 days) from 0 to 10, where 0 indicated the worst job performance and 10 indicated the best. The presenteeism score was the scale score × 10 (range: 0–100), with higher scores indicating no lack of performance. The cutoff score for tending to be absent due to mental health issues was 40, with scores of 40 or less indicating greater presenteeism [32].

Demographic data were collected, namely, gender, age, height, weight, smoking status (which was dichotomized into a non-smoker and a current smoker), alcohol consumption (which was dichotomized into non-consumption and ≥1 time/month), marital status, childcare (preschool child), family caregiving, and academic history (which was dichotomized into senior high school or less [i.e., 12 years or less] and some college or higher [i.e., more than 12 years]).

Psychosocial data were also collected, namely, annual household income (which was grouped into <4 million, 4–8 million, and ≥8 million yen), industry type (which was dichotomized into secondary and tertiary industries), occupation (which was dichotomized into managerial and non-managerial workers), frequency of remote working from home per week during the previous month (which was dichotomized into ≤4 days per week and ≥5 days per week [fully remote]), COVID-19 involvement (which was dichotomized into no involvement and involvement), and perceived stress in the workplace. Perceived stress in the workplace was determined from a single question derived from the Special Survey on Industrial Safety and Health [33] that asked if participants had experienced something that had given them anxiety, worry, or distress at work or in their occupational life, for which there were four response options: no, rather no, rather yes, and yes (which were dichotomized into yes or no).

### 2.3. Analyses

The participants were categorized into 10 groups based on whether their DRP was <11, 11–12, 13–14, 15, or ≥16 h and whether their sleep duration was <6 or ≥6 h, with a group for every DRP–sleep duration combination. A 15-h DRP, which corresponded to a standard 8-h work shift with a 1-h lunch break, was set as an adequate DRP, and <11-h DRP, which was referred to as QR, was set as a short DRP. Although previous studies divided the DRP into 1-h intervals [3,8,34], 11, 12, 13, and 14 h of DRP was grouped into 11–12 and 13–14 h as this study specifically focused on short DRPs (<11 h) and adequate DRPs (15 h). The National Sleep Foundation recommends 7–9 h of sleep per day and does not recommend <6 h of sleep for adults aged 26–64 years [35]; however, as few Japanese workers slept more than 7 h a day (15.3%) [36], the sleep durations were divided into <6 and ≥6 h of sleep, which were set as short sleep duration and normal sleep duration, respectively.

Chi-squared tests on the 10 groups and two-way analysis of variance (ANOVA) of DRP (five groups) and sleep duration (two groups) were conducted to compare the demographic and psychosocial statuses as well as the DRPs and sleep durations.

Logistic regression analyses were conducted to estimate the odds ratios (ORs) with a 95% confidence interval (CI) for the joint association of DRP and sleep duration in the 10 groups with sleep difficulties (AIS ≥ 6), poor mental health (K6 scale ≥ 13), and presenteeism (WHO-HPQ, presenteeism score ≤ 40). These cutoff scores were based on previous studies [25,28,32]. The crude ORs (model 1) were estimated and adjusted for the basic demographic factors, namely, gender, age, body mass index, smoking status, alcohol consumption, education, marital status, childcare, and family caregiving (model 2), as well as the psychosocial factors, namely, annual household income, industry type, occupation, remote working from home, perceived stress in the workplace, and COVID-19 involvement (model 3). The 15-h DRP and ≥6-h sleep group, consisting of participants with adequate DRP and normal sleep duration, was set as the reference group. The ORs and 95% CIs for the <11-h DRP and <6-h sleep group (QR and short sleep duration group), <11-h DRP and ≥6-h sleep group (QR and normal sleep duration group), and 15-h DRP and <6-h sleep group (adequate DRP and short sleep duration group) were focused on to examine hypotheses 1, 2, and 3. The statistical significance level was set at α = 0.05.

All statistical analyses were conducted using IBM SPSS Statistics version 23.0 for Microsoft Windows (IBM Corp., Armonk, NY, USA).

## 3. Results

**Figure 1:**
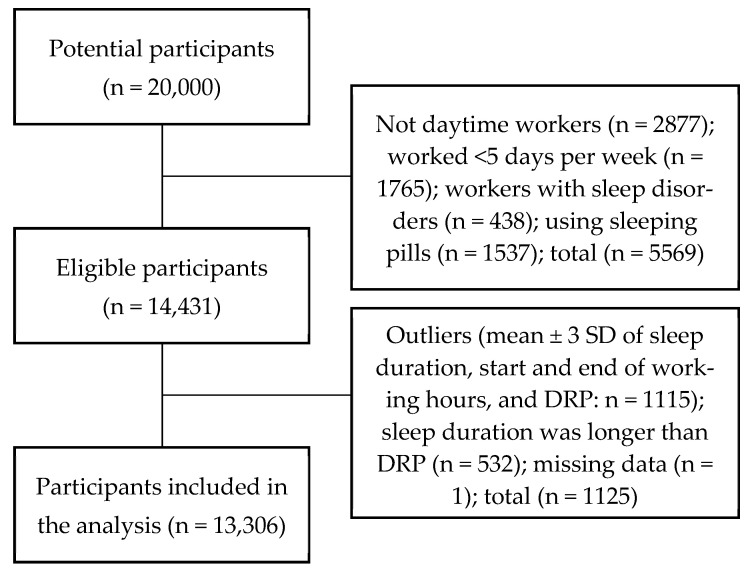
Participant enrolment. SD, standard deviation; DRP, daily rest period.

Cross-sectional data from 20,000 participants were first evaluated. The data of 5569 participants were excluded because they were shift or night workers (*n* = 2877), were working less than five days a week (*n* = 1765), were being treated for sleep disorders (*n* = 438), and/or had used sleeping pills within the previous year (*n* = 1537). Another 1125 participants with outlying values (±3 standard deviations [SD] from the mean of the eligible participants [*n* = 14,431] for sleep duration, start and end of working hours, and/or DRP: *n* = 1115), inconsistent data (sleep durations longer than the DRP, *n* = 532), and/or missing data (*n* = 1) were also excluded. The final sample comprised 13,306 Japanese daytime workers (Figure 1).

The participants were categorized into 10 unequally sized groups based on their DRP and sleep duration (Table 1).

Table 1 presents the participant characteristics in each group. The mean DRP and sleep duration were 14.7 and 6.4 h. The chi-squared tests revealed significant group differences in gender, smoking status, alcohol consumption, marital status, childcare, family caregiving, education, annual household income, occupation, industry type, remote working, perceived stress in the workplace, and COVID-19 involvement (all *p* < 0.01). The two-way ANOVA revealed that DRP and sleep duration (all *p* < 0.001) had significant interactions.

Table 2 presents the incidence ratios and logistic regression analysis results of the joint association of DRP and sleep duration with sleep difficulty, mental health, and presenteeism. The logistic regression analyses for sleep difficulty revealed that the <11-h DRP and <6-h sleep group (OR = 4.45, 95% CI = 2.83–7.01: model 1), <11-h DRP and ≥6-h sleep group (OR = 1.81, 95% CI = 1.19–2.77: model 1), and 15-h DRP and <6-h sleep group (OR = 2.53, 95% CI = 2.19–2.92: model 1) had higher ORs than the reference group.

The logistic regression analyses for mental health revealed that the <11-h DRP and <6-h sleep group (OR = 3.04, 95% CI = 1.79–5.15: model 1), <11-h DRP and ≥6-h sleep group (OR = 2.05, 95% CI = 1.12–3.72: model 1), and 15-h DRP and <6-h sleep group (OR = 1.93, 95% CI = 1.56–2.40: model 1) had higher ORs than the reference group.

The logistic regression analyses for presenteeism revealed that the <11-h DRP and <6-h sleep group (OR = 2.35, 95% CI = 1.47–3.77: model 1) as well as the 15-h DRP and <6-h sleep group (OR = 1.39, 95% CI = 1.16–1.67: model 1) had higher ORs than the reference group.

These significant associations remained after adjusting for the demographic and psychosocial factors (models 2 and 3). Of the 10 groups, the <11-h DRP and <6-h sleep group was found to be the most likely to have sleep difficulties, poor mental health, and presenteeism.

## 4. Discussion

This study investigated the joint association of DRP and sleep duration with worker health and productivity in Japanese daytime workers. It was found that a short DRP (specifically, QR), short sleep duration, or both were associated with high risks of experiencing sleep difficulties, having poor mental health, and presenteeism and that the ORs for the combination of QR and short sleep duration were the highest among all combinations.

The finding that QR and short sleep duration combined associated most strongly with poor outcomes confirms Hypothesis 1. Although previous studies have reported that each of these factors are associated with poor worker health and productivity [3,4,5,8,9,10,11,12,13,14], this study found that worker health and productivity suffered the most when a short DRP and short sleep duration occurred simultaneously.

Although the combination of QR and short sleep duration was associated with poor worker health and productivity, the dominant factor in this association may be short sleep duration. Workers with short sleep durations (<6 h) had sleep difficulties regardless of whether they had long or short DRPs, but those with normal sleep duration (>6 h) had sleep difficulties only when they had short DRP of <11–12 h. Similar results were obtained for mental health. Furthermore, workers with short sleep durations exhibited greater presenteeism when they had a DRP of <11–15 h; however, workers with normal sleep durations did not show presenteeism regardless of whether they had a long or short DRP. These results suggest that short sleep duration was the dominant factor in the combined association with negative worker outcomes.

The combination of QR and normal sleep duration was associated with high risks of sleep difficulties and poor mental health, which partially supports Hypothesis 2. Moreover, this suggests that even if workers get a normal amount of sleep, their sleep quality and mental health may deteriorate if QR is demanded of them. Previous studies have reported that short DRP (QR) has negative effects on worker health and productivity [3,4,5]. However, the DRP contains the period used for sleep, and workers with a shorter DRP tended to show shorter sleep duration [3]. Since short sleep duration also has negative effects on worker health and productivity [8,9,10,11,12,13,14], the effects of short DRP and sleep duration may have been confounded in previous studies. The hypothesis in the current study, however, focused on workers with a short DRP and normal sleep duration, and the results showed that even if workers have normal sleep duration, their health can deteriorate when their employers require QR. In other words, this study found that short DRP alone, without a short sleep duration, is negatively associated with worker health.

The cause of this association between QR (despite an adequate sleep duration) and poor worker health is as yet a matter of speculation. However, previous studies have demonstrated that pre-sleep cognitive arousal degrades sleep quality and can result in a prolongation of sleep latency [37] and decreasing electroencephalogram (EEG) delta power density [38]. It may be that workers who get a normal amount of sleep during QR have a short interval from the end of work to bedtime, and this causes high arousal level prior to sleep onset, damaging their sleep quality and mental health. This could account for the need for an adequate DRP as well as a normal sleep duration to ensure good worker health.

The combination of an adequate DRP and short sleep duration was associated with high risks of sleep difficulties, poor mental health, and presenteeism, which supports Hypothesis 3. Previous studies have reported that a short sleep duration has negative effects on worker health and productivity [8,9,10,11,12,13,14]. However, as mentioned above, previous studies potentially confounded DRP with sleep duration. The hypothesis focused on workers with a short sleep duration and adequate DRP and found that even workers with an adequate DRP had poorer health and productivity outcomes if they had short sleep duration. In other words, a short sleep duration alone, without a short DRP, is associated with poor worker health and productivity. These findings suggest that even if a certain DRP is ensured by the “work interval system” in Japan or the “daily rest period” in the EU working time directive, worker health and productivity may deteriorate if workers do not have a sleep duration of greater than 6 h. The National Sleep Foundation also recommends 7–9 h and not <6 h of sleep for adults aged 26–64 years [35]. Therefore, workers who have <6 h of sleep may need to prioritize sleep during DRP.

Of the workers with QR (*n* = 176), 50% had short sleep durations, and 50% had normal sleep durations. The DRP includes sleep opportunities and other leisure time and essential life activities, such as commuting, eating, and bathing. As it is difficult to reduce these essential life activities, short DRPs may lead to short sleep opportunities and short leisure times. A previous cross-sectional study reported that although DRP was correlated with both sleep duration and leisure time, the correlation between DRP and sleep duration was significantly weaker than that between DRP and leisure time [3]. Furthermore, another longitudinal study reported that in a group with DRPs shortened by more than 2 h, their leisure time decreased by more than 2 h, but their sleep duration decreased by less than 1 h [4]. Therefore, the discrepancy between decreasing sleep opportunities and leisure time may have made it possible to ensure an adequate sleep duration, that is, workers may prioritize a certain sleep duration over leisure time if they have shorter DRPs.

This survey was conducted during the COVID-19 pandemic in Japan, and there were group differences in involvement in the present study. The pandemic has increased mental health problems in the global population, and workers involved in countering the pandemic (e.g., healthcare workers), in particular, have been shown to have worse mental health-related outcomes, such as levels of depression [39] and insomnia [40]. Preventing mental health problems in workers who are directly involved in fighting COVID-19 is very important, and it will be necessary to consider measures to address this issue in the future.

Notably, workers who had a “normal” sleep duration (defined here as ≥6 h) reported sleep difficulties. The mean sleep duration was less than 7 h in every group. Therefore, these workers’ sleep duration may be not have been sufficient, and some may have been experiencing sleep difficulties. In addition, sleep quality can be affected by factors, such as DRP [3,5] and timing of sleep [41]. Those of these factors that were not examined in this study might cause sleep difficulties, so further studies are needed to clarify this issue.

This study had several limitations. First, the data were collected using a web survey, which means that there may have been sampling bias. Second, all data used in this study were self-reported; therefore, further studies using objective data are warranted. Third, the present study focused on daytime non-shift workers; therefore, whether similar results would be obtained from shift and/or night workers is unclear. Fourth, data were not collected on how the workers used their DRP, that is, the actual balance between sleep duration, leisure time, and essential life activities during the DRP was unclear. Finally, cross-sectional studies cannot provide information about causal relationships, and there is the possibly of reverse causation, residual confounding, and chance. This study was part of WELWEL, which is an ongoing prospective study; therefore, there will be opportunities in the future to examine these relationships longitudinally, and this may solve some of these problems.

## 5. Conclusions

This study investigated the joint association of DRP and sleep duration with worker health and productivity in Japanese daytime workers and found cross-sectional associations between both factors and all the worker outcomes. Therefore, ensuring adequate DRP and normal sleep duration is the best way to achieve good worker health and productivity.

## Figures and Tables

**Table 1:** Participant characteristics in each group.

Daily Rest Period (Hours):	Total	<11	<11	11–12	11–12	13–14	13–14	15	15	≥16	≥16	
Sleep Duration (hours):	Total	<6	≥6	<6	≥6	<6	≥6	<6	≥6	<6	≥6	*p* Value ^a^
*n*:	13,306	88	88	329	738	919	3788	911	4547	320	1578	
Gender, female, *n* (%)	5824 (43.8)	12 (13.6)	25 (28.4)	64 (19.5)	108 (14.6)	329 (35.8)	1269 (33.5)	464 (50.9)	2071 (45.5)	265 (82.8)	1217 (77.1)	<0.001
Age, years, mean (SD)	41.8 (10.1)	41.0 (10.1)	39.5 (8.9)	44.5 (9.4)	40.8 (9.9)	44.7 (9.4)	40.8 (10.3)	44.8 (9.6)	41.0 (10.2)	46.1 (8.9)	42.1 (9.7)	0.649
Body mass index, mean (SD)	22.2 (3.6)	23.1 (3.7)	22.3 (3.9)	22.9 (3.9)	22.9 (3.3)	22.9 (4.1)	22.3 (3.4)	22.5 (4.0)	22.1 (3.6)	21.6 (3.8)	21.4 (3.4)	0.204
Smoking status, current smoker, *n* (%)	2806 (21.1)	22 (25.0)	23 (26.1)	101 (30.7)	206 (27.9)	208 (22.6)	851 (22.5)	206 (22.6)	929 (20.4)	53 (16.6)	207 (13.1)	<0.001
Alcohol consumption, ≥1 time/week, *n* (%)	7210 (54.2)	49 (55.7)	44 (50.0)	189 (57.4)	443 (60.0)	519 (56.5)	2242 (59.2)	455 (49.9)	2393 (52.6)	126 (39.4)	750 (47.5)	<0.001
Marital status, married, *n* (%)	7104 (53.4)	55 (62.5)	52 (59.1)	191 (58.1)	470 (63.7)	496 (54.0)	2110 (55.7)	443 (48.6)	2202 (48.4)	182 (56.9)	903 (57.2)	<0.001
Childcare, yes, *n* (%)	2374 (17.8)	17 (19.3)	20 (22.7)	69 (21.0)	166 (22.5)	121 (13.2)	705 (18.6)	113 (12.4)	776 (17.1)	54 (16.9)	333 (21.1)	<0.001
Family caregiving, yes, *n* (%)	583 (4.4)	1 (1.1)	1 (1.1)	21 (6.4)	34 (4.6)	50 (5.4)	151 (4.0)	57 (6.3)	175 (3.8)	17 (5.3)	76 (4.8)	<0.001
Education, ≥12 years, *n* (%)	10,300 (77.4)	74 (84.1)	68 (77.3)	277 (84.2)	599 (81.2)	728 (79.2)	3102 (81.9)	664 (72.9)	3492 (76.8)	202 (63.1)	1094 (69.3)	<0.001
Annual household income, *n* (%)												<0.001
<4 million yen	3560 (26.8)	16 (18.2)	19 (21.6)	59 (17.9)	105 (14.2)	209 (22.7)	841 (22.2)	271 (29.7)	1367 (30.1)	121 (37.8)	552 (35.0)	
4–8 million yen	6105 (45.9)	37 (42.0)	42 (47.7)	138 (41.9)	374 (50.7)	403 (43.9)	1804 (47.6)	417 (45.8)	2048 (45.0)	129 (40.3)	713 (45.2)	
≥8 million yen	3641 (27.4)	35 (39.8)	27 (30.7)	132 (40.1)	259 (35.1)	307 (33.4)	1143 (30.2)	223 (24.5)	1132 (24.9)	70 (21.9)	313 (19.8)	
Occupation, managerial workers, *n* (%)	1544 (11.6)	20 (22.7)	13 (14.8)	94 (28.6)	140 (19.0)	165 (18.0)	572 (15.1)	86 (9.4)	399 (8.8)	7 (2.2)	48 (3.0)	<0.001
Industry type, secondary industry, *n* (%)	3946 (29.7)	28 (31.8)	22 (25.0)	110 (33.4)	271 (36.7)	328 (35.7)	1264 (33.4)	263 (28.9)	1351 (29.7)	46 (14.4)	263 (16.7)	<0.001
Remote working, ≥5 days per week, *n* (%)	856 (6.4)	10 (11.4)	7 (8.0)	11 (3.3)	46 (6.2)	46 (5.0)	272 (7.2)	44 (4.8)	322 (7.1)	5 (1.6)	93 (5.9)	<0.001
Perceived stress in the workplace, yes, *n* (%)	8631 (64.9)	67 (76.1)	62 (70.5)	265 (80.5)	519 (70.3)	670 (72.9)	2535 (66.9)	634 (69.6)	2733 (60.1)	214 (66.9)	932 (59.1)	<0.001
COVID-19 involvement, involvement, *n* (%)	2289 (17.2)	23 (26.1)	19 (21.6)	76 (23.1)	174 (23.6)	183 (19.9)	735 (19.4)	136 (14.9)	691 (15.2)	42 (13.1)	210 (13.3)	<0.001
Sleep duration, hours, mean (SD)	6.4 (1.0)	4.8 (0.5)	6.4 (0.7)	5.0 (0.5)	6.6 (0.7)	5.0 (0.5)	6.8 (0.7)	5.0 (0.5)	6.8 (0.7)	5.0 (0.4)	6.8 (0.8)	<0.001
Daily rest period, hours, mean (SD)	14.7 (1.5)	9.6 (1.0)	9.5 (1.4)	12.0 (0.6)	12.2 (0.5)	13.9 (0.6)	14.0 (0.6)	15.2 (0.2)	15.2 (0.2)	17.0 (1.2)	17.0 (1.2)	<0.001

^a^ Results of the chi-squared tests or the interactions for the two-way ANOVAs.

**Table 2:** Joint association of daily rest period (DRP) and sleep duration with sleep difficulty, mental health, and presenteeism (*n* = 13,306).

	Incidence	Odds Ratio (95% Confidence Interval)		
DRP, Sleep Duration	Ratio (%)	Model 1 ^a^	Model 2 ^b^	Model 3 ^c^
Sleep difficulties (Athens insomnia scale ≥ 6)				
<11 h, <6 h (*n* = 88)	68.2	**4.45 (2.83–7.01)**	**4.94 (3.13–7.81)**	**4.49 (2.78–7.27)**
<11 h, ≥6 h (*n* = 88)	46.6	**1.81 (1.19–2.77)**	**1.87 (1.22–2.86)**	**1.71 (1.09–2.67)**
11–12 h, <6 h (*n* = 329)	56.5	**2.70 (2.16–3.39)**	**3.08 (2.45–3.88)**	**2.51 (1.97–3.19)**
11–12 h, ≥6 h (*n* = 738)	42.0	**1.51 (1.28–1.76)**	**1.62 (1.38–1.91)**	**1.45 (1.22–1.72)**
13–14 h, <6 h (*n* = 919)	49.3	**2.02 (1.75–2.33)**	**2.24 (1.94–2.59)**	**1.99 (1.71–2.32)**
13–14 h, ≥6 h (*n* = 3788)	33.9	1.07 (0.97–1.17)	**1.10 (1.00–1.21)**	1.00 (0.91–1.11)
15 h, <6 h (*n* = 911)	54.9	**2.53 (2.19–2.92)**	**2.71 (2.34–3.14)**	**2.60 (2.22–3.03)**
15 h, ≥6 h (*n* = 4547)	32.5	1.00	1.00	1.00
≥16 h, <6 h (*n* = 320)	52.2	**2.27 (1.81–2.85)**	**2.41 (1.91–3.04)**	**2.39 (1.87–3.06)**
≥16 h, ≥6 h (*n* = 1578)	31.9	0.97 (0.86–1.10)	0.96 (0.84–1.09)	0.98 (0.86–1.11)
Poor mental health (K6 ≥ 13)				
<11 h, <6 h (*n* = 88)	20.5	**3.04 (1.79–5.15)**	**3.63 (2.11–6.25)**	**2.98 (1.70–5.22)**
<11 h, ≥6 h (*n* = 88)	14.8	**2.05 (1.12–3.72)**	**2.20 (1.20–4.05)**	**1.98 (1.05–3.72)**
11–12 h, <6 h (*n* = 329)	12.8	**1.73 (1.23–2.43)**	**2.18 (1.54–3.09)**	**1.72 (1.20–2.45)**
11–12 h, ≥6 h (*n* = 738)	9.1	1.18 (0.90–1.55)	**1.38 (1.04–1.82)**	1.21 (0.91–1.61)
13–14 h, <6 h (*n* = 919)	12.7	**1.72 (1.38–2.15)**	**2.04 (1.63–2.56)**	**1.75 (1.39–2.21)**
13–14 h, ≥6 h (*n* = 3788)	7.6	0.96 (0.82–1.13)	1.02 (0.87–1.20)	0.92 (0.78–1.09)
15 h, <6 h (*n* = 911)	14.1	**1.93 (1.56–2.40)**	**2.14 (1.72–2.67)**	**1.93 (1.54–2.42)**
15 h, ≥6 h (*n* = 4547)	7.8	1.00	1.00	1.00
≥16 h, <6 h (*n* = 320)	9.4	1.22 (0.83–1.81)	1.36 (0.91–2.02)	1.20 (0.80–1.81)
≥16 h, ≥6 h (*n* = 1578)	7.5	0.96 (0.78–1.20)	0.97 (0.77–1.20)	0.98 (0.78–1.23)
Presenteeism (WHO-HPQ short form presenteeism score < 40)				
<11 h, <6 h (*n* = 88)	28.4	**2.35 (1.47–3.77)**	**2.22 (1.38–3.58)**	**1.94 (1.19–3.16)**
<11 h, ≥6 h (*n* = 88)	21.6	1.63 (0.98–2.73)	1.52 (0.91–2.56)	1.40 (0.82–2.38)
11–12 h, <6 h (*n* = 329)	26.7	**2.17 (1.67–2.80)**	**2.29 (1.76–2.97)**	**1.94 (1.48–2.53)**
11–12 h, ≥6 h (*n* = 738)	18.7	**1.36 (1.11–1.67)**	**1.28 (1.04–1.57)**	1.17 (0.95–1.45)
13–14 h, <6 h (*n* = 919)	20.1	**1.49 (1.25–1.79)**	**1.64 (1.36–1.97)**	**1.47 (1.22–1.77)**
13–14 h, ≥6 h (*n* = 3788)	15.4	1.08 (0.95–1.22)	1.05 (0.93–1.19)	0.99 (0.87–1.12)
15 h, <6 h (*n* = 911)	19.0	**1.39 (1.16–1.67)**	**1.57 (1.30–1.90)**	**1.45 (1.20–1.75)**
15 h, ≥6 h (*n* = 4547)	14.4	1.00	1.00	1.00
≥16 h, <6 h (*n* = 320)	12.8	0.87 (0.62–1.22)	1.14 (0.81–1.60)	1.05 (0.74–1.48)
≥16 h, ≥6 h (*n* = 1578)	11.7	**0.78 (0.66–0.93)**	0.90 (0.75–1.07)	0.90 (0.75–1.08)

Reference (1.00) = daily rest period of 15 h with sleep duration ≥ 6 h. WHO-HPQ = World Health Organization Health and Work Performance Questionnaire. ^a^ Crude model. ^b^ Adjusted for gender, age, body mass index, smoking status (0: non-smoker, 1: current smoker), alcohol consumption (0: non-consumption, 1: more than once per week), education (0: <12 years, 1: ≥12 years), marital status (0: unmarried, 1: married), childcare (0: no, 1: yes), family caregiving (0: no, 1: yes). ^c^ Model 2 + annual household income (<4 million yen, 4–8 million yen, ≥8 million yen), industry type (0: tertiary industry, 1: secondary industry), occupation (0: non-managerial workers, 1: managerial workers), remote working from home (0: <5 day/week, 1: ≥5 day/week), perceived stress at the workplace (0: no, 1: yes), and COVID-19 involvement (0: no involvement, 1: involvement). Significant odds ratios (*p* < 0.05) and their 95% confidence interval values are presented in boldface.

## Data Availability

Data available on request from the corresponding author.
